# The impact of advanced footwear technology on running performance and pacing in world marathon majors

**DOI:** 10.3389/fphys.2026.1800107

**Published:** 2026-05-13

**Authors:** Yuya Maruo, Kensuke Takezawa

**Affiliations:** 1 Department of Health Design, Showa Women’s University, Setagaya, Tokyo, Japan; 2 Division of Physical and Health Education, Setsunan University, Osaka, Japan

**Keywords:** advanced footwear technology, endurance, long-distance running, marathon, pacing

## Abstract

**Introduction:**

This study investigated the impact of Advanced Footwear Technology (AFT) on marathon performance and pacing strategies within the World Marathon Majors (WMM). While AFT is known to improve running economy (RE), it remains unclear whether AFT enhances overall running velocity across the race or primarily alters pacing strategies.

**Methods:**

We analyzed the top 10 performances from the Berlin, Chicago, London, New York City, and Tokyo marathons across two periods: the non-AFT era (2013–2015) and the standardized AFT era (2022–2025). There were 221 male athletes and 211 female athletes. To examine differences in pacing patterns between groups, linear mixed-effects models were applied.

**Results:**

AFT significantly improved total finish time for both men (approximately 1.7%) and women (approximately 4.0%). In men, LMM revealed significantly higher performance during the AFT era; however, a decline in running velocity over distance was observed in both groups. In women, the LMM showed a significantly greater decline in running velocity with distance in the AFT era than in the non-AFT era, after accounting for race and athlete variability.

**Discussion:**

This study demonstrated that AFT is associated with improved marathon performance. In men, AFT is associated with higher running velocities but does not appear to substantially alter pacing patterns. In women, AFT leads to faster initial velocity followed by a steeper late-race decline, indicating a more aggressive pacing strategy and premature deceleration. These findings highlight that optimal initial pacing has become increasingly critical in the AFT era.

## 1 Introduction

The World Marathon Majors (WMM) represents the pinnacle of elite road racing, consistently attracting the highest caliber of athletes. Analyzing data from this global circuit provides a robust framework for evaluating elite performance under competitive, real-world conditions. Recently, marathon performance has been transformed by Advanced Footwear Technology (AFT)—defined as a performance-enhancing combination of lightweight, resilient midsole foams and rigid longitudinal plates (Frederick, 2022). AFT emerged in prototype form around 2016; however, its use in elite competition and widespread commercial availability began from 2017 onwards. While early projects like Breaking2 (2017) and the INEOS 1:59 Challenge (2019) demonstrated the physiological potential of AFT under optimized, non-compliant conditions, it is of critical interest to investigate the impact of AFT on actual competitive race dynamics, specifically regarding finish times and 5-km split pacing.

Previous studies have confirmed that AFT enhances running performance in both half-marathon and marathon events (Bermon et al., 2021; Langley and Langley, 2024; Rodrigo-Carranza et al., 2021, 2022; Willwacher et al., 2024). Specifically, Rodrigo-Carranza et al. (2022) observed significant improvements across the 10km, half-marathon, and marathon, with the most substantial gains in the longer distances. Quantifying this impact, Langley and Langley (2024) analyzed 971 elite performances from 2012 to 2021, finding a statistically significant 1.0% increase in velocity—equivalent to an approximately 79-second reduction in marathon time. Similarly, Willwacher et al. (2024) examined world top-100 rankings from 2010 to 2022, reporting that post-AFT performance enhancements were particularly pronounced among female athletes and in long-distance disciplines. Collectively, these findings suggest that AFT has significantly enhanced running economy (RE), directly contributing to the surge of record-breaking performances since the late 2010s. Alongside evolutions in training and tactics, AFT has emerged as a critical factor in the recent advancement of world-class distance running records.

Furthermore, previous studies have explored the biomechanical and physiological underpinnings of AFT. It is well-established that AFT improves RE compared to conventional footwear (Barnes and Kilding, 2019; Day and Hahn, 2020; Healey and Hoogkamer, 2022; Hébert-Losier et al., 2022; Hunter et al., 2019; Hoogkamer et al., 2017, 2018, 2019; Joubert et al., 2024; Ortega et al., 2021; Rodrigo-Carranza et al., 2024; Shorten, 2024; Steele et al., 2025). For example, Hoogkamer et al. (2018) reported a 4% RE improvement over conventional shoes, while Joubert et al. (2024) observed a 2% gain in AFT track spikes. These benefits are characterized by significantly lower oxygen uptake (Hunter et al., 2019) and attenuated neuromuscular fatigue (Steele et al., 2025). Shorten (2024) attributes these performance gains to increased longitudinal bending stiffness, lightweight design, and enhanced cushioning, rather than shifts in endogenous physiological capacity. However, as these findings derive predominantly from laboratory settings, the extent to which AFT influences pacing and deceleration points in actual marathon races remains to be fully elucidated.

To achieve optimal performance, middle- and long-distance athletes must strategically manage their energy expenditure until the finish line. Extensive research has identified various pacing strategies (Angus, 2014; Edwards and Polman, 2013; Foster et al., 1994; Gibson et al., 2006; Renfree and Gibson, 2013; Tucker, 2009; Tucker and Noakes, 2009), emphasizing that marathon success depends on selecting an initial pace aligned with one’s physiological capacity (Renfree and Gibson, 2013). Adopting an unsustainable early speed leads to significant late-race deceleration, resulting in performances well below an athlete’s potential. This selection is even more critical in WMM races where pacers are utilized, as optimized pacing may offer a “shortcut” to further gains, even at the world-record level (Angus, 2014). Furthermore, AFT-mediated enhancements in RE may alter an athlete’s perception of effort, potentially decoupling perceived exertion from actual physiological strain. In elite racing, where the margin for error is minimal, AFT enables runners to adopt aggressive initial velocities that were previously prohibitive. However, improved efficiency does not inherently expand total energy stores or lipid oxidation capacity at extreme intensities. This raises a critical question: does AFT truly delay fatigue, or does it merely enable an aggressive pacing strategy that accelerates the transition toward catastrophic metabolic failure? Understanding whether these performance gains are sustained or result in premature deceleration is essential for evaluating AFT’s impact on competitive dynamics. Investigating shifts in initial speed and the occurrence of late-race deceleration will provide valuable insights into evolving race strategies in the AFT group.

This study aimed to elucidate the impact of AFT on the running performance and pacing in WMM. For this end, we compiled the records of the marathon. Records from 2022 to 2025 were defined as the AFT era, while those from 2013 to 2015 were defined as the non-AFT era. Although AFT was introduced in 2016, this study focuses on the four-year period from 2022 to 2025. Considering the impact of COVID-19 and variability in the use of AFT, we defined the period from 2022 to 2025 as the AFT era. During this period, the footwear regulations established by World Athletics were fully implemented and stably enforced (World Athletics, 2026). We calculated the mean and variance and compared the statistical differences between groups. We compared 5-km split running velocity to investigate in which race segments running velocity increased or decreased with the use of AFT. There are few studies that have examined these effects using data from actual marathon races. Therefore, we tested the following hypotheses. If AFT effectively delays the onset of fatigue and improves RE without inducing over-pacing, the running velocity during the latter of the marathon will remain stable, resulting in a significantly reduced deceleration compared to the non-AFT era. In this case, a significant interaction between group and split running velocity would be expected, showing a “flatter” pacing profile in the AFT era. On the one hand, if the improvement in RE encourages athletes to adopt an aggressive initial velocity that exceeds their aerobic capacity, the overall race speed will increase, but the fundamental pacing breakdown in the late stages will persist or even occur earlier. In this case, we expected an interaction between AFT era and distance, such that the AFT era would exhibit a decline in running velocity or pace earlier than the non-AFT era.

## 2 Methods

### 2.1 Participants

Top 10 performances in each WMM (Berlin, Boston, Chicago, London, New York City, Tokyo) were obtained from Web results. Records from the Boston Marathon were excluded because its down-hill course design does not comply with World Athletics standards. Data were categorized into two groups. According to the footwear regulations established by World Athletics, from 2022 onwards the regulatory framework governing footwear was fully implemented and stably operated, resulting in standardized technical criteria for approved shoes (World Athletics, 2026). Therefore, the period from 2022 to 2025 was defined as the “AFT era”. Given that the aim of this study was to evaluate the impact of recent developments in AFT, all available data up to 2025 were included in order to reflect the current state of WMM. Although AFT shoes were officially released in 2017, prototypes were identified as early as 2016 (Mason et al., 2024). Furthermore, the 2012 New York City Marathon was cancelled due to a hurricane and no data were recorded. Consequently, the period from 2013 to 2015 was defined as the “non-AFT era”. The year 2016 was excluded as a transitional period in the adoption of AFT, which resulted in an asymmetry in the comparison windows. A linear mixed model (LMM) was employed, as described below, which is appropriate for handling unbalanced data structures. All races included in this study were officially sanctioned by World Athletics (WA) and conducted in accordance with WA regulations. Accordingly, course-specific characteristics were assumed to be within the acceptable limits defined by these regulations. The data for the 8th-place finisher in the 2025 Chicago marathon were not available because the split times had not been publicly released. A total of 699 performances were analyzed. To properly account for repeated appearances of the same athletes across different years and races, a unique identifier (ID) was assigned to each athlete. There were 221 male athletes and 211 female athletes. This study involved the analysis of publicly available data so that individual informed consent was not necessary. This study was approved by Local Ethics Committee (Kenrinshin 2023-56).

### 2.2 Data analysis

Descriptive statistics were calculated for each group. We calculated the mean and standard deviation of finish times and running velocities for each 5-km segment and the final 2.195 km. To examine differences in pacing patterns between groups, LMM were applied. Running velocity was modeled as a function of distance, AFT era, and their interaction. Distance was treated as a continuous variable. AFT era (AFT vs. non-AFT) was included as a fixed effect. The interaction between distance and AFT era was included to assess whether the change in performance across distance differed between groups. To account for repeated observations within athletes and potential differences between races, athlete and races were included as random intercepts. All models were fitted using restricted maximum likelihood (REML), and statistical significance was assessed using Satterthwaite’s approximation for degrees of freedom. In addition, analyses were repeated using normalized performance values to minimize inter-individual differences in baseline performance. Normalization was performed by expressing performance at each distance relative to each athlete’s overall mean performance. The same model structure was applied to the normalized data. All statistical analyses were executed using RStudio for Windows (Posit team, 2025).

## 3 Results

### 3.1 Descriptive statistics

**Table 1** showed the start time, temperature, and humidity. For men’s race, total finish time were 2:06:57 ± 2:40.7 in AFT era and 2:09:08 ± 2:55.7 in non-AFT era. For women’s race, total finish time were 2:22:00 ± 4:04.4 in AFT era and 2:27:38 ± 4:18.4 in non-AFT era.

**Table 1:** Start time, temperature, and humidity for each race.

Event name	AFT era			
	2022	2023	2024	2025
Tokyo	March 3, 9:10 11.3°C, 30% Simultaneous	March 5, 9:10 8.5°C, 48% Simultaneous	March 3, 9:10 9.6°C, 22% Simultaneous	March 2, 9:10 14.0°C, 41% Simultaneous
London	October 23, F9:00, M9:40 13.7°C, 89% Separated	April 23, F9:00, M9:30 11.3°C, 75% Separated	April 23, F9:25, M10:00 8.1°C, 58% Separated	April 27, F9:10, M9:35 12°C, 76% Separated
Berlin	September 25, 9:15 12°C, 78% Simultaneous	September 24, 9:15 13°C, 76% Simultaneous	September 29, 8:50 9°C, 81% Simultaneous	September 21, 9:15 19°C, 78% Simultaneous
Chicago	October 9, 7:30 7°C, 60% Simultaneous	October 8, 7:30 8°C, 68% Simultaneous	October 13, 7:30 15°C, 77% Simultaneous	October 12, 7:30 11°C, 85% Simultaneous
New York	November 6, W8:40, M9:05 22°C, 79% Separated	November 5, W8:40, M9:05 10°C, 64% Separated	November 3, W8:35, M9:05 5°C, 58% Separated	November 2, W8:35, M9:05 7°C, 69% Separated
Event name	non-AFT era			
	2013	2014	2015	
Tokyo	February 24, 9:10 4.6°C, 39% Simultaneous	February 23, 9:10 4.0°C, 38% Simultaneous	February 22, 9:10 8.0°C, 34% Simultaneous	
London	April 13, F9:00, M9:45 8°C, 90% Separated	April 13, F10:15, M10:00 11°C, 53% Separated	April 18, F9:05, M9:20 8°C, 75% Separated	
Berlin	September 29, 8:45 7°C, 93% Simultaneous	September 28, 8:45 11°C, 86% Simultaneous	September 27, 9:00 10°C, 80% Simultaneous	
Chicago	October 13, 7:30 9°C, 72% Simultaneous	October 12, 7:30 8°C, 70% Simultaneous	October 11, 7:30 12°C, 70% Simultaneous	
New York	November 3, F9:10, M9:40 11°C, 50% Separated	November 2, F9:10, M9:40 7°C, 48% Separated	November 1, F9:20, M9:50 15°C, 62% Separated	

F, female race; M, Male race.

### 3.2 Liner mixed model analysis

**Figure 1:**
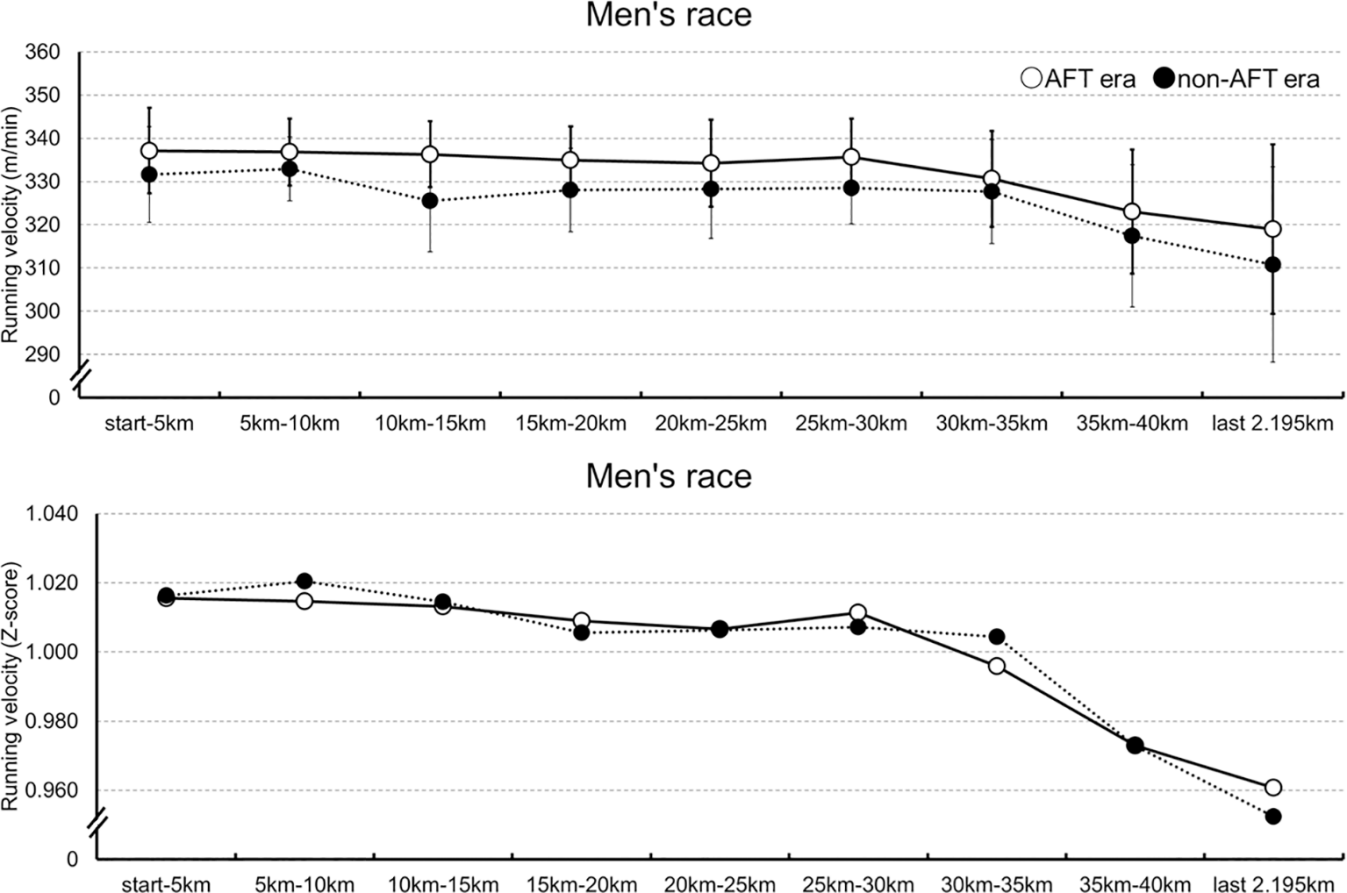
The men’s running velocity for each segment in both groups.

**Figure 1** depicts the men’s marathon running velocity and normalized running velocity for each segment in both groups. For men’s running velocity, a linear mixed-effects model was fitted with athlete and race included as random intercepts to account for inter-individual and inter-race variability. There was a significant main effect of distance (*β* = -0.42, SE = 0.05, *t*(2948) = -8.6, *p* <.001, 95% CI [-0.52, -0.33]). A significant main effect of AFT era was also observed (*β* = -6.39, SE = 1.04, *t*(1561) = -6.13, *p* <.001, 95% CI [-8.44, -4.35]). The interaction between distance and AFT group was not significant (*β* = 0.01, SE = 0.03, *t*(2948) = 0.10, *p* = .92, 95% CI [-0.06, 0.07]).

For men’s normalized running velocity, a linear mixed-effects model was fitted to the normalized performance data with athlete and race included as random intercepts. The variance components for athlete and race were estimated to be approximately zero, indicating minimal variability attributable to these factors after normalization. There was a significant main effect of distance (*β* = -0.01, SE = 0.01, *t*(3146) = -8.34, *p* <.001). The main effect of AFT era was not significant (*β* = 0.01, SE = 0.01, *t*(3146) = 1.22, *p* = .22). The interaction between distance and AFT group was also not significant (*β* = -0.01, SE = 0.01, *t*(3146) = -1.37, *p* = .17).

**Figure 2:**
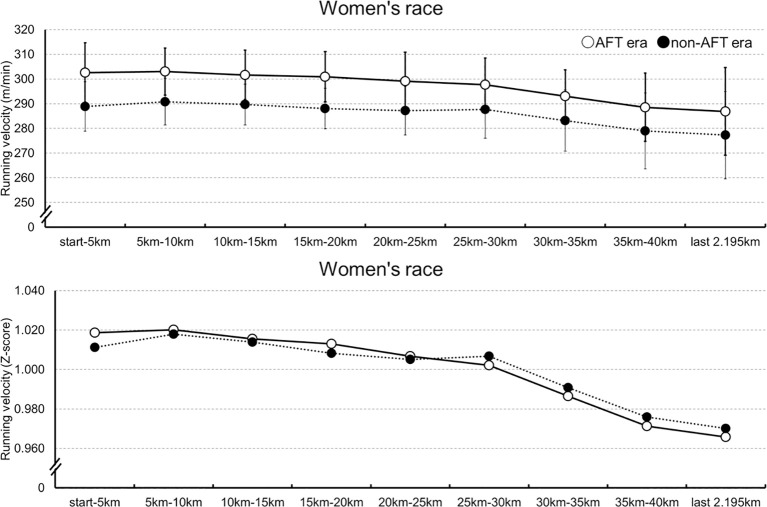
The women’s running velocity for each segment in both groups.

**Figure 2** depicts the women’s marathon running velocity and normalized running velocity for each segment in both groups. For women’s running, velocity, a linear mixed-effects model was fitted with athlete and race included as random intercepts to account for inter-individual and inter-race variability. There was a significant main effect of distance (*β* = -0.54, SE = 0.04, *t*(2909) = -12.64, *p* <.001, 95% CI [-0.63, -0.46]). A significant main effect of AFT era was also observed (*β* = -12.98, SE = 1.17, *t*(740) = -11.10, *p* <.001, 95% CI [-15.27, -10.69]). In addition, a significant interaction between distance and AFT era was found (*β* = 0.109, SE = 0.028, *t*(2909) = 3.84, *p* <.001, 95% CI [0.05, 0.16]).

For women’s normalized running velocity, a linear mixed-effects model was fitted to the normalized performance data with athlete and race included as random intercepts. The variance components for athlete and race were estimated to be approximately zero, indicating minimal variability attributable to these factors after normalization. There was a significant main effect of distance (*β* = -0.01, SE = 0.01, *t*(3137) = -13.23, *p* <.001). A significant main effect of AFT group was also observed (*β* = -0.01, SE = 0.01, *t*(3137) = -3.12, *p* = .001). In addition, a significant interaction between distance and AFT era was found (*β* = 0.01, SE = 0.01, *t*(3137) = 3.49, *p* <.001).

## 4 Discussion

The present study aimed to elucidate the impact of AFT on the running performance and pacing in WMM. The primary findings of this study were that running velocity increased in both sexes following the introduction of AFT. Furthermore, when pacing was compared using normalized data, pacing for women’s marathon in the AFT era showed a greater decrease in velocity as the race progressed. In contrast, for men’s marathon, no significant differences in pacing were observed between the non-AFT and AFT eras. While the finding that running velocity increased is consistent with previous research (Bermon et al., 2021; Langley and Langley, 2024; Rodrigo-Carranza et al., 2021, 2022; Willwacher et al., 2024), the discovery that pacing patterns in women’s marathons differed depending on the use of AFT is novel and noteworthy.

The present findings align with previous research indicating that marathon performance among world-class elite athletes has improved significantly following the introduction of AFT in 2019 (Bermon et al., 2021; Langley and Langley, 2024; Rodrigo-Carranza et al., 2021, 2022; Willwacher et al., 2024). Finish times improved by approximately 1.7% for men’s marathon and 4.0% for women’s marathon, values that closely align with the 2–5% enhancement in RE reported in previous laboratory-based longitudinal studies (Hoogkamer et al., 2018; Joubert et al., 2024; Langley et al., 2023; Mason et al., 2024). This suggests that AFT contributes to higher running velocity. Muniz-Pardos et al. (2021) suggested that improvements in performance are attributable not so much to enhancements in physiological capacity as to improvements in running mechanics, that is, technical factors. In addition, it has been suggested that female athletes may benefit more from AFT. Compared with men, women typically have a lower body mass, which may influence energy efficiency, and a higher stride frequency, potentially increasing the number of ground contacts and, consequently, the opportunities to benefit from AFT (Langley et al., 2023; Mason et al., 2024). The overall performance gain appears to be primarily driven by a significantly faster pace.

To examine running velocity and pacing in detail, we analyzed running velocity across 5-km segments and the final 2.195 km using LMM. In the women’s marathon, the LMM showed that performance was higher in the AFT era than in the non-AFT era. In addition, a significant interaction between distance and AFT era indicated that the pattern of change across distance differed between groups, with a greater decline in running velocity observed in the AFT era. This suggests that races in the AFT era were characterized by a more aggressive pacing strategy, leading to a larger reduction in running velocity in the latter stages of the race. Although a greater decline in running velocity was observed in the AFT era, finish times remained superior compared to the non-AFT era. This indicates that the more aggressive pacing strategy does not adversely affect overall performance. To examine pacing patterns rather than running velocity per se, we also conducted an LMM using z-score–normalized data. This normalization largely removed between-athlete and between-race variability, thereby isolating pure differences in pacing patterns. Importantly, the interaction effect remained significant after normalization, indicating that this difference reflects a difference in pacing itself, rather than a performance-level difference. Athletes in the AFT era appear to adopt a more aggressive pacing strategy, which consequently leads to a greater relative decline in the latter stages of the race.

This “substantial deceleration” suggests a difference in pacing: the mechanical running advantages provided by AFT may reduce perceived effort, encouraging athletes to adopt fast early-race pace. One possible explanation is that since AFT does not increase total endogenous glycogen storage, such aggressive early pacing could be associated with an earlier onset of pace down. Furthermore, this tendency might be further influenced by situational factors. Female runners have reported to be more susceptible to competitors (Renfree and Gibson, 2013). In the context to WMM races, where female runners may compete in proximity to male runners, this could contribute to a tendency toward overpacing.

The LMM for men’s marathon revealed that the decline in running velocity with increasing distance was significantly both in the AFT era and in the non-AFT era. This finding suggests that performance decreased as the race progressed. In addition, running velocity across the entire race was higher in the AFT era than in the non-AFT era, indicating superior overall performance in the AFT era. Importantly, even after statistically controlling for race differences across Tokyo, Berlin, Chicago, London, and New York City, significant main effects of group and distance were observed. Therefore, these results cannot be explained solely by course or race differences, indicating that the AFT era is associated with higher running velocities, while both groups exhibit a decline in pace toward the latter stages of the race. The normalized performance decreased as the race progressed, suggesting no overall difference in normalized performance between groups. The pattern of performance change over distance did not differ between the AFT and non-AFT groups. Given these results, in men’s WMM marathons, AFT is associated with higher running velocities but does not appear to substantially alter pacing patterns.

Our findings suggest that, particularly in women, the selection of an appropriate initial velocity has become more critical than ever in the AFT era. Although the observed effects were statistically significant, the effect sizes were relatively small. However, in elite marathon, even small differences can be practically meaningful, as race outcomes are often determined by narrow margins. Therefore, these findings may still have important implications for pacing strategy. Previous studies have demonstrated that the positive pace strategy and even pace strategy are the better strategy to achieve a record (Angus, 2014; Renfree and Gibson, 2013). Even in the non-AFT era, selecting an appropriate initial velocity commensurate with an individual’s capacity was considered a fundamental requirement for marathon success (Renfree and Gibson, 2013). Renfree and Gibson (2013) observed that elite female runners often overpaced by reacting to competitors, leading to ‘poor decisions’ that exceeded their physiological limits. AFT spike shifts the pacing for perceived exertion (Bertschy et al., 2025). However, caution is required when comparing middle-distance track spikes to marathon footwear, as the metabolic demands and mechanical requirements differ significantly between these disciplines. Although perceived exertion cannot be directly equated between spike shoes and marathon shoes, the same underlying technology has been implemented in both; therefore, female athletes and coaches may need to recalibrate their pacing strategies to avoid premature pacing breakdown. The earlier deceleration observed in women’s marathon may suggest that the traditional 30–35 km “wall” (Berndsen et al., 2020; Buman et al., 2008) may occur earlier when initial pacing is too aggressive under AFT conditions. Future training approaches may need to focus not only on improving mechanical efficiency but also on enhancing aerobic capacity and glycogen availability to sustain the higher velocity enabled by AFT.

It should be noted that our study included some limitations. First, as this study focused on time-oriented WMM races, the results may not generalize to tactical championship races (e.g., Olympics), where ranking takes precedence over finish time and pacing dynamics are influenced by tactical surges rather than optimal efficiency. Second, the specific timeframe used for comparison should be considered. The depth of elite fields may have differed between the 2013–2015 and 2022–2025 periods. This choice of periods was necessitated by the emergence of AFT prototypes in 2016 (Mason et al., 2024) and the cancellation of the 2012 New York City Marathon due to a hurricane. As AFT continues to evolve, the optimal selection of comparison periods merits further consideration in future research. Third, regarding the presence and quality of pacemakers, consistent official data were not available across all events, making it difficult to include these as individual covariates in the model. However, by using an LMM with ‘Race’ as a random effect, we have attempted to comprehensively control for between-race environmental variability, including course characteristics, weather conditions, and the inherent pacing tendencies of each event. In future research, it may be useful to establish inclusion criteria based not only on ranking but also on finishing time. Future research should examine whether AFT has altered the dynamics of such high-stakes tactical racing.

## Data Availability

The original contributions presented in the study are included in the article/supplementary material. Further inquiries can be directed to the corresponding author.
